# Perspectives on how mucosal immune responses, infections and gut microbiome shape IgA nephropathy and future therapies

**DOI:** 10.7150/thno.49778

**Published:** 2020-09-15

**Authors:** Jia-Wei He, Xu-Jie Zhou, Ji-Cheng Lv, Hong Zhang

**Affiliations:** Renal Division, Peking University First Hospital; Peking University Institute of Nephrology; Key Laboratory of Renal Disease, Ministry of Health of China; Key Laboratory of Chronic Kidney Disease Prevention and Treatment (Peking University), Ministry of Education, Beijing, 100034, People's Republic of China.

**Keywords:** IgA nephropathy, mucosal immune response, infection, gut microbiome, therapy

## Abstract

Infections have been considered to play a critical role in the pathogenesis of IgA nephropathy (IgAN) because synpharyngitic hematuria is a common feature in IgAN. However, how infections participate in this process is still debated. More recent studies have also revealed that the alteration of the gut microbiome exerts a profound effect on host immune responses, contributing to the etiology or progression of autoimmunity. Considering IgA as the first line of defense against bacterial and viral antigens, this review evaluates the relationships among intestinal infections, gut microbiome, and IgA for a better understanding of the pathogenesis of IgAN. Moreover, as a prototype of IgA immunity, we provide detailed clarification of IgAN pathogenesis to shed light on other diseases in which IgA plays a role. Finally, we discuss potential therapies focusing on microbes and mucosal immune responses in IgAN.

## Introduction

Immunoglobulin A (IgA) nephropathy (IgAN) is the most common primary glomerulonephritis in the world, primarily affecting young people aged between 20 and 40 years' old 1. In particular, 20%-40% of patients with IgAN will progress to end-stage renal disease within 20 years of disease onset 2. Despite an increased understanding of disease pathogenesis and improved treatment options, IgAN remains a major cause of mortality and morbidity. Currently, the diagnosis of IgAN can only be confirmed through pathological assessment via invasive kidney biopsy. Moreover, its corresponding therapies depend primarily on nonspecific inhibitors of the renin-angiotensin system (RAS) and immunosuppressants, which are not uniformly effective.

**Figure 1:**
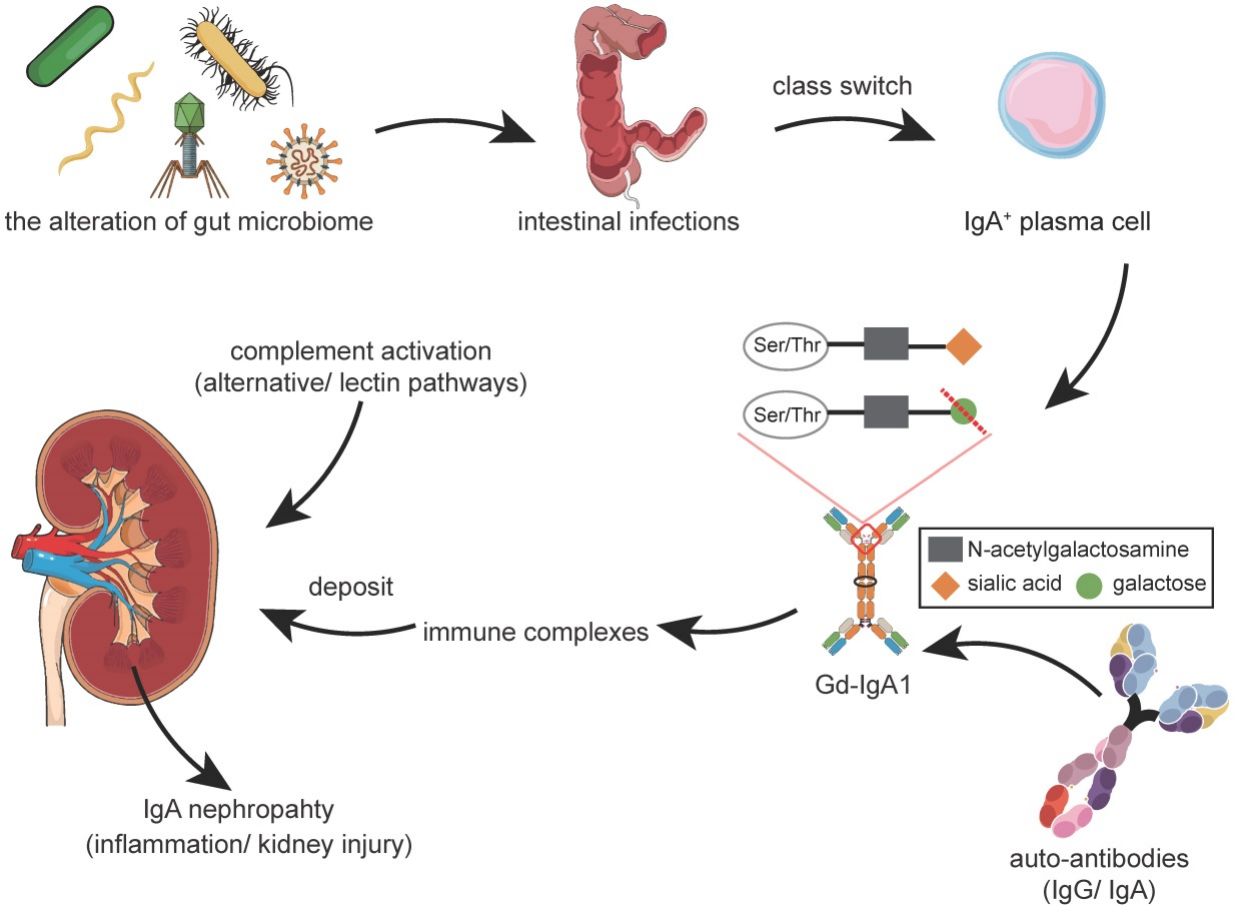
** Abnormal Mucosal Immune Responses and Development of IgAN.** The alteration of the gut microbiome is considered to be a novel factor involved in the pathogenesis of IgAN. Dysbiosis ultimately results in changes of microbial functions, including changes in biochemical processes and fermentation productions. Loss of the immune equilibrium could make the host susceptible to intestinal infections. Intestinal infections prime the class switch of B-cells class switch, leading to the overproduction of Gd-IgA1. Excess Gd-IgA1 acts as an autoantigen and will be bound by autoantibodies, such as IgG or IgA. These immune complexes deposit in the glomerular mesangium, including mesangial proliferation. A series of downstream pathways, including the complement system, will be activated, leading to inflammation and glomerular injury. Abbreviations: Gd-IgA1: galactose-deficient IgA1; Ser: serine; Thr: threonine.

Currently, IgAN is regarded as an immune-mediated disease characterized by the deposition of polymeric and hypogalactosylated IgA1 (Gd-IgA1) in glomerular mesangium. Compared with healthy controls, patients with IgAN have been reported to exhibit elevated serum levels of IgA and Gd-IgA1 3,4. The incomplete glycosylation of IgA1 has also been shown to further act as an autoantigen, inducing the production of autoantibodies 5-8. By the likely help of IgA receptors, the resultant deposition of these Gd-IgA1-containing immune complexes (ICs) in the glomerular mesangium has been reported to cause cellular proliferation and overproduction of the extracellular matrix, cytokines, and chemokines, leading to glomerular injury 9,10. Complement activation is also known to be critical in the pathogenesis of IgAN, which can take place directly on IgA1/IgA1-containing ICs in circulation or after their deposition in the mesangial area 11 (**Figure 1**).

However, there are still some controversies surrounding the etiology and pathogenesis of IgAN 12,13. It has been suggested that selective IgA1 hyperresponsiveness plays a crucial role in the pathogenesis of IgAN. However, the exact production site of the Gd-IgA1 remains controversial. In addition, the corresponding regulatory mechanism of this increased circulating IgA1 has not been addressed. Emerging data supports that undue mucosal immune responses instead of bone marrow might be the first perpetrators.

This review mainly evaluates the relationships among intestinal infections, gut microbiome, and IgA for a better understanding of the pathogenesis of IgAN. Moreover, we provide detailed clarification of IgAN pathogenesis and discuss potential therapies focusing on gut microbes and mucosal immune responses in IgAN.

## Immunoglobulin A (IgA)

Briefly, IgA, the second most abundant isotype in the circulation, is known to mainly consist of monomers derived from bone marrow plasma cells, whereas secretory IgA is synthesized as dimers by local plasma cells before being transported to mucosal surfaces through epithelial cells by the polymeric immunoglobulin receptor. In serum, IgA is mainly monomeric, belonging to the IgA1 subclass with rapid catabolism (half-life: 3-6 d) 14. Moreover, IgA is the most glycosylated form of immunoglobulins, with carbohydrates representing about 6% of its content 15. Unlike IgA2, the heavy chains of IgA1 molecules are known to contain a unique insertion in the hinge-region segment with a high content of serine and threonine residues. These residues are the sites of attachment of up to 5 O-linked glycan chains consisting of N-acetylgalactosamine with a β1,3-linked galactose and sialic acids. Sialic acid can also be attached to N-acetylgalactosamine by an α2,6 linkage 16. Although IgA is derived from the adaptive immune system, they do have innate-like recognition properties. Polyreactive IgA seems to be a product of the coevolution of host and microbiota for the maintenance of symbiotic homeostasis. The peculiar property of IgA has been shown to facilitate its binding to different structurally diverse antigens 17. Currently, there have been no studies directly elaborating the relationship between polyreactive IgA and IgAN.

In normal situations, our host is protected from infections through the production of IgA, which is particularly predominant at intestinal mucosa. Together with mucus and antimicrobial peptides, they are known to form the first line of defense 18,19. Patients with selective IgA deficiency show increased frequencies of upper respiratory or gastrointestinal infections 20. Upon infections, increased synthesis of IgA has been shown to help eliminate pathogens and maintain homeostasis at mucosal surfaces 21,22. However, mucosal infections, if persistent, might lead to overproduction of IgA, which might become pathogenic 23,24. Increased serum levels of IgA or increased circulating levels of IgA1-IgG ICs constitute one of the main features of IgAN, with altered glycosylation favoring the self-aggregation of IgA1 25.

Apart from invading pathogens, dysbiosis of the gut microbiome has been suggested to also contribute to the overproduction of IgA 13,26. In supporting this, studies showed that the microbial characteristics among patients with IgAN and healthy controls were different. More specifically, higher percentages of the *Ruminococcaceae*, *Lachnospiraceae*, *Eubacteriaceae, Streptococcaeae, Escherichia-Shigella*, *Hungatella*, or *Eggerthella* genera/species could be observed in patients with IgAN 27,28. Moreover, patients with IgAN presented significantly higher intestinal permeability, which was related to increased proteinuria, microhematuria, and serum levels of IgA when compared with healthy controls 14. Thus, it is necessary to clarify the crosstalks among mucosal immune responses, infections, and the gut microbiome in the development of IgAN. A deeper understanding of the dysregulation of IgA from the perspectives of intestinal infections and gut microbiome should be of pivotal significance in understanding the pathogenesis of IgAN and diseases in which IgA is involved, such as IgA vasculitis, ankylosing spondylitis, Sjögren's syndrome, alcoholic liver cirrhosis, celiac disease, inflammatory bowel disease (IBD), and dermatitis herpetiformis. Besides, it might aid the development of disease-specific therapies, as well as the identification of noninvasive disease-specific biomarkers in the future.

## The mucosal origin of Gd-IgA1

The majority of circulating IgA1 is known to be monomeric, heavily O-galactosylated, and considered to be derived from bone marrow-residing plasma cells. Bone marrow, especially the red bone marrow, is one of the critical components of the lymphatic system. It is the primary lymphoid organ generating lymphocytes from immature hematopoietic progenitor cells. Accordingly, bone marrow is an important source of plasma IgA in healthy human beings. It has been demonstrated that human bone marrow mononuclear cells can secrete a significant amount of *de novo* synthesized IgA *in vitro* 29. However, these kinds of monomeric IgA1 have been shown to be different from those found in the glomerular mesangial area and not considered to be pathogenic in IgAN 30,31. The rest of the circulating IgA1 is mostly dimeric and poorly O-galactosylated 32. At present, the origin of the circulatory Gd-IgA1 remains controversial. Plasma cells in the mucosal immune system are known to synthesize IgA1 that is predominantly dimeric, and galactose-deficient 33. The secreted Gd-IgA1 has been shown to be the same as that in the circulatory systems. Thus, it has been speculated that circulatory Gd-IgA1 might be the result of mucosal-derived plasma cells.

The mucosal immune system includes both inductive and effector sites. Amongst all, inductive sites are the positions where naïve B-cells are exposed to antigens. Tonsils, as well as Peyer's patches in the small intestine, are common inductive sites in humans. The interaction of mucosal-derived antigens with B-cells has been reported to prime its activation through T-cell-dependent and T-cell-independent pathways, inducing an isotype class-switch from IgG/IgM to IgA 34. The immune induction sites of mucosa-associated lymphoid tissue lack afferent lymphatics, but do have efferent lymphatics, which is different from those in other peripheral lymphoid tissues. Activated B-cells are known to translocate to regional lymph nodes through efferent lymphatics. Then, these cells enter into the circulatory systems and subsequently translocate to the lamina propria of the inductive and the remote mucosal membranes, in which they act as effector cells 35. In the intestine, those IgA plasmablasts will mature into IgA-secreting plasma cells in the lamina propria, and the synthetic polymeric IgA will then bind to polymeric immunoglobulin receptors on the basolateral surface of mucosal epithelial cells. After internalization, they will be transcytosed into vesicles to the mucosal apical surface. Finally, following cleavage of the secretory component, secretory IgA will be released to the lumen 36. However, it has been postulated that some mucosal-derived plasma cells might mis-home to the bone marrow during lymphocyte trafficking, but its mechanism remains unclear. Faulty expression of surface homing receptors on lymphocyte subsets or defective expression of mucosal chemokines and homing counterreceptors on mucosal vascular endothelium might also account for this presumption 37-40.

Some patients with IgAN will develop visible hematuria within 12 to 24 h after upper respiratory tract and gastrointestinal infections. Because gross hematuria has been shown to often follow an episode of upper tract respiratory infection, IgAN is also called synpharyngitic glomerulonephritis. As IgA is known to have a significant role in mucosal immune responses, the concurrence of visible hematuria is thought to be due to an abnormal mucosal immune response to the overproduction of IgA1 41. It has been suggested that stimulations caused by pathogenic microbes are recognized by pattern recognition receptors, such as Toll-like receptors (TLRs). These stimulations have been reported to promote the secretion of B-cell-activating factor (BAFF) and enhance lymphocytic infiltration, the expression of histocompatibility complex class II molecules on B-cells, and IgA synthesis. Persistent and excessive activation of TLRs might eventually favor the overproduction of IgA1/Gd-IgA1 and O-glycan-specific antibodies 23. Clinically, a proportion of comorbidities of IgAN in chronic mucosal infections (streptococcus, staphylococcus), IBD, and celiac disease have indicated the existence of a common mucosal mechanism. Thus, the above evidence supports that the mucosal immune system might be the main production site of Gd-IgA1.

In human bodies, the mucosal immune system is known to primarily consist of lymphoid tissues that are distributed in the skin, oral cavity, respiratory tract, urinary tract, and the gastrointestinal tract. However, the role of the individual mucosa, such as nasopharynx-associated lymphoid tissue (NALT) or gut-associated lymphoid tissue (GALT) in the pathogenesis of IgAN needs further verification. Moreover, defining the part of the mucosal immune system that might be mainly responsible for the production of Gd-IgA1 remains a controversy.

Clinically, a high percentage of patients with IgAN suffer from respiratory infections, such as tonsillitis and pharyngitis. These infections might leave them vulnerable to pronounced hematuria, proteinuria, or the deterioration of the renal function 13. Thus, some researchers believe that NALT has a critical influence over the onset and progression of IgAN 42-44. Amongst all, tonsils, which are part of the lymphatic system, have been considered to be a source of pathogenic Gd-IgA1 45,46. B-cells can be activated and proliferate in germinal centers in the tonsil. Germinal centers are critical sites where B memory cells are created and secretory IgA is synthesized. The number of IgA^+^ plasma cells has been reported to be elevated in the tonsils of patients with IgAN compared with that in disease controls with recurrent tonsillitis 47,48. Besides, it has been demonstrated that plasma cells can become memory (long-lived) plasma cells, producing antibodies for a long time 49. Some of these memory plasma cells might disseminate into the bone marrow, in which they synthesize and release Gd-IgA1 into the circulatory system. In recent years, many studies have also focused on the microbial ecology of the upper respiratory tract in patients with IgAN. For instance, the *Haemophilus parainfluenzae*, oral indigenous bacterium, was shown to stimulate the proliferation of tonsillar mononuclear cells and the production of IgA *in vitro* 50. Comprehensive microbiome analysis of tonsillar crypts revealed that similar patterns of bacteria existed in both IgAN and recurrent tonsillitis. In particular, it was found that *Prevotella spp., Fusobacterium spp., Sphingomonas spp.*, and *Treponema spp.* were predominant in the above populations. Thus, these findings indicated that these microbes were of importance in the development of IgAN 51. Besides, the variant rs2412971, intronic in *HORMAD2*, was also shown to be tightly associated with tonsillectomy. The risk allele for tonsillectomy corresponded to an increased risk of IgAN 52. Thus, it was suggested that NALT might be critical in the development of IgAN, and tonsillectomy might act as one promising therapeutic approach. Some studies have revealed that tonsillectomy could decrease the serum or salivary secretory levels of IgA, especially in children 53. There have been concerns about whether tonsillectomy would cause significant immune deficiency 54. But some researchers believed that tonsillectomy would not cause immune deficiencies. Although tonsillectomy could decrease the serum levels of IgA, these treatments have been reported to be clinically insignificant 55,56. It was shown that after tonsillectomy, the lymphocyte levels of CD8^+^ T increased, whereas the levels of B-cells returned to normal 57. Overall, the role of tonsils in the pathogenesis of IgAN or the effects of tonsillectomy needs further verification.

In contrast, some patients with IgAN have been shown to have clinical associations with intestinal diseases, such as IBD or coeliac disease. Being the most extensive and essential part of the mucosal immune system, the intestinal immune system is known to be an important site of recognition of antigens and immune cell activation 9,58. Thus, in recent years, some experts have held the view that GALT might also be an important site for the production of Gd-IgA1, affecting the development of IgAN. Furthermore, it was evidenced that Gd-IgA1 was also present in patients with ulcerative colitis 59. However, these findings were confined to sporadic cases and hence needed verification in large cohorts. More importantly, curative therapies of intestinal diseases have shown promising results in treating IgAN. For example, sulfasalazine and 5-aminosalicylic have been shown to inhibit the spontaneous secretion of IgA by intestinal mononuclear cells 60,61. Budesonide has been used to treat patients with asthma, inflammatory bowel disease, and allergic rhinitis, and its enteric-coated sustained-release capsule was demonstrated to decrease the level of proteinuria in patients with IgAN, indicating a reduced risk of future progression to end-stage renal disease 62. Similarly, in celiac disease, gluten-free diets have been reported to decrease the levels of proteinuria and mitigate immune disorders in patients with IgAN, showing reduced levels of antiendomysium antibodies 63. Thus, further research is needed to determine the role of GALT in the pathogenesis of IgAN.

## The role of mucosal infections in IgA/Gd-IgA1 immunity

The propensity of mucosal infections coinciding with hematuria has led to the hypothesis that defects in the regulation of the local IgA response might trigger IgAN. Currently, at least 3 hypotheses have been proposed to address the role of mucosal infections in the pathogenesis of IgAN: specific pathogens, chronic and persistent exposure to mucosal infections, and alterations of the gut microbiome 23.

**Figure 2:**
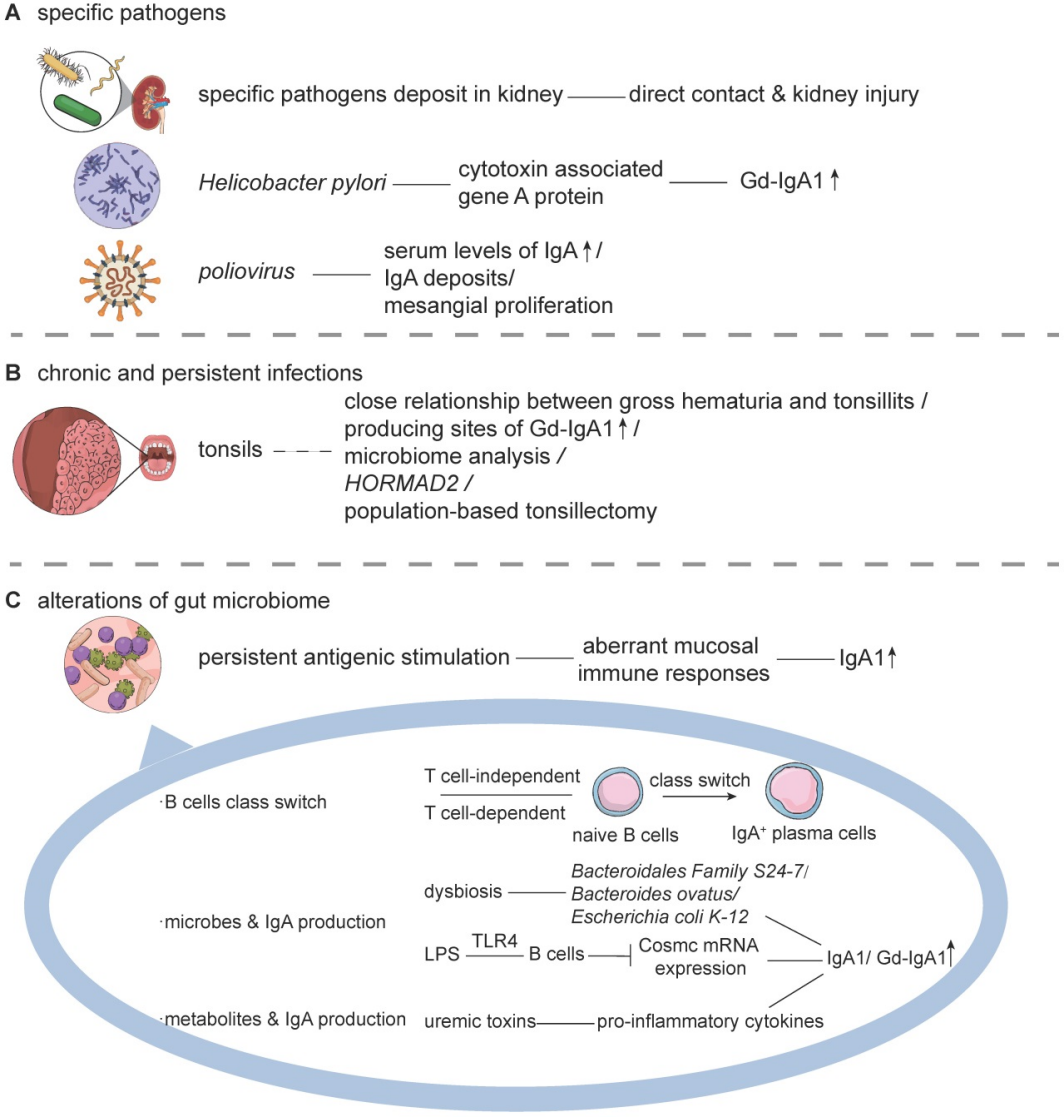
** Mucosal Infections and Immune Responses.** Mucosal infections are implicated in the pathogenesis of IgAN through at least 3 hypotheses. **A.** The hypothesis of specific pathogens. Specific pathogens are believed to be involved in the initiation and progression of IgAN. Several pathogens could be directly detected in renal tissue. Several other pathogens, such as *Helicobacter pylori* and *poliovirus,* affect the production of IgA1 and the hypogalactosylation process. **B.** The hypothesis of chronic and persistent infections. The occurrence of tonsillitis is believed to be related to IgAN. Clinically, there is a close relationship between upper respiratory infections and hematuria. Morphological analyses have shown that tonsils are important producing sites of Gd-IgA1. Additional evidence includes the microbiome analysis of tonsillar crypts in IgA nephropathy, genetic association analysis (*HORMAD2*), and the effectiveness of population-based tonsillectomy. Thus, chronic and persistent tonsillitis might be involved in disease pathogenesis but require further verification. **C.** Intestinal infections caused by the alterations of the gut microbiome have been gradually recognized as a critical inducer in the pathogenesis of IgAN. Persistent antigenic stimulation causes aberrant mucosal immune responses, affecting the class switch of B-cells and the overproduction of IgA. Abbreviations: Gd-IgA1: galactose-deficient IgA1; LPS: lipopolysaccharide; TLR: toll-like receptor.

The first hypothesis considers that specific pathogens are involved in the initiation and progression of IgAN. A series of evidence has demonstrated their direct participation in disease pathogenesis (**Figure 2A**). For instance, some pathogens, such as *Human Cytomegalovirus*, *Adeno and Herpes simplex viral*, *Haemophilus parainfluenzae*, *Staphylococcus aureus,* and* Epstein-Barr virus* have been detected in renal tissues from patients with IgAN, in accordance with IgA deposits 64-68. In concert, these specific pathogens might interact with binding sites in the glomerulus, inducing kidney injury. Except for the direct deposits, some pathogens have also been involved in the production and glycosylation of IgA1. For instance, it has been observed that infection by *Helicobacter pylori* was correlated with elevated levels of Gd-IgA1 in patients with IgAN 69,70, and its cytotoxin associated gene A protein stimulated the production of IgA1 in a dose- and time-dependent manner. This promoted the underglycosylation of IgA1, which was partly attributed to the downregulation of β1,3-galactosyltransferase and its Cosmc chaperone in DAKIKI cells, an immortalized IgA1 production cell line 71. Experiments in BALB/C mice immunized by a *poliovirus* vaccine revealed the presence of higher serum levels of IgA, predominant IgA and C3 deposits, and mesangial proliferation in renal histopathology 72. Likewise, the *Sendai virus*, or *respiratory syncytial virus,* was also observed to have the capacity to promote the release of inflammatory cytokines, such as interleukin-6 (IL-6), and prostaglandin E2 by mesangial cells, and to induce mesangial proliferation, similar to the renal pathological features of IgAN 73,74. However, this hypothesis has not been widely accepted. Those with opposing views have pointed out that several of the pathogens mentioned above could also be detected in patients with other glomerular diseases, that is, *Cytomegalovirus* in membranous nephropathy, minimal change disease, or membranoproliferative glomerulonephritis; *Haemophilus parainfluenzae* in non-IgA glomerulonephritis and systemic lupus erythematosus 64,66. Some have refuted that these findings were documented only in sporadic cases. In addition, various pathogen detection techniques or different ways of collecting biopsy tissue might have also contributed to these inconsistencies. Unpurified DNA in the polymerase chain reaction might introduce contamination by polymerase-inhibiting substances, and the possibility of nonspecific binding could not be excluded.

The second hypothesis believes the chronic and persistent exposure to mucosal infections to be closely associated with IgAN (**Figure 2B**). Tonsillitis is one such representative example. Some Japanese studies have indicated that tonsillectomy might be beneficial among some patients with IgAN in reducing proteinuria, hematuria, and increasing renal functions 75,76. Tonsillectomy or tonsillectomy plus immunosuppressants has been demonstrated to induce clinical remission in some early-stage patients with IgAN and might protect long-term renal survival 77-79. Accordingly, it was shown that partial patients with IgAN receiving tonsillectomy plus steroid pulse therapy had improved proteinuria, hematuria, and renal function. The serum levels of Gd-IgA1, IgA/C3 ratio, along with some urinary inflammatory markers, such as IL-6, intercellular adhesion molecule-1, monocyte chemotactic protein-1, or kidney injury molecule-1, have been reported to be significantly declined after this kind of therapy 45,80. However, the curative effects of tonsillectomy have not been shown to be consistent among similar studies, indicating high disease heterogeneity among different races or populations 81,82. It has been further observed that the activation of innate immunity via TLRs, ubiquitin-proteasome, as well as the prooxidative milieu, were not affected by tonsillectomy in patients with IgAN 83. Apart from the no apparent influence on innate immunity, related studies from Western countries, such as the "VALIGA" study, had not demonstrated the benefits of tonsillectomy in Caucasian populations 84. Thus, in the updated 2020 KDIGO guidelines, the performance of tonsillectomy was not advised as a treatment for IgAN in Caucasians, suggesting an ethnic or environmental difference.

The third hypothesis holds that alterations of the gut microbiome could affect both systemic or local immune responses. Under normal conditions, the host and gut microbiome maintain homeostasis, with the host offering a balanced immune response to microbes. Respectively, innate and adaptive immune systems are known to form a biochemical barrier between the host and gut microbiome. However, in cases where the barrier is broken down, dysbiosis might occur, leading to a severe inflammatory response. Aberrant immune responses have been shown to lead to increased infiltration of proinflammatory cells, such as neutrophils, dendritic cells, Th1, or Th17 cells 85. Meanwhile, alterations of the gut microbiome could increase the antigen load and epithelial TLR recognition, which might thus promote the class switch of B-cells and the overproduction of IgA 23,86. This means that the ecosystem could shift to a state of dysbiosis, involving the overgrowth of otherwise underrepresented or potentially harmful bacteria 87. The persistent antigenic challenge might also lead to alterations of intestinal permeability, subclinical inflammatory state, or mucosa-associated lymphoid tissue activation 88. For instance, the ectopic colonization of *Klebsiella spp.* has been associated with the persistent activation of the immune system. Stains of *Klebsiella*, such as *Klebsiella pneumoniae 2H7* (especially the outer membrane protein X), have been reported to function as potent inducers of Th1 cells, contributing to the accumulation of TH1 cells observed in mice 89,90. The colonization of *Candida albicans* (such as the expression of adhesins and invasins on the cell surface) has been shown to cause persistent antigenic stimulation by promoting the release of IL-17 or IFN-γ by Th17 cells 91,92. Relevant studies have additionally suggested that the colonization of gut microbes could affect the intestinal immune status. Based on these, it was demonstrated that raising IgAN experimental model mice in a germ-free environment or using broad-spectrum antibiotics to eliminate intestinal pathogens could decrease the serum levels of IgA1 and IgA1-containing ICs, and prevent IgA1 mesangial deposition and glomerular inflammation 93,94.

## The role of gut microbiota in IgA/Gd-IgA1 immunity

Gut microbiota and their derived metabolites have been suggested to have a significant impact on immune homeostasis 95 (**Figure 2C**). It has been estimated that the intestinal tract harbors up to 100 trillion microbes 26. These gut microbiota maintain the integrity of the epithelial barrier and shape the intestinal immune system, balancing host defense with microbial metabolites, components, and attachment to host cells 96. Concomitantly, intestinal mucosa contains a large number of different immune cells that participate in the maintenance of a healthy microbiota community, as well as reinforce epithelial barrier functions 97. Mounting evidence has underpinned the essential roles of the gut microbiome as critical regulatory elements in host immune responses.

The gut microbiome is known to be involved in the class switch of B-cells. Recent evidence has indicated that microbial infections in the intestine promote the class switch of naive B-cells to IgA antibody-secreting cells, through both T-cell-independent (TLR ligation) and T-cell-dependent (cytokine-mediated) pathways. More specifically, intestinal infections have been reported to promote the production of IL-6, IL-10, transforming growth factor β (TGF-β), B-cell activating factor (BAFF), and TNF superfamily member 13 (TNFSF13, also known as APRIL) by intestinal epithelial, dendritic, and stromal cells, which have an essential role in stimulating the class switch of B-cells via the T-cell-independent pathway 86,98. APRIL is a member of the tumor necrosis factor superfamily and has been shown to mediate the CD40-independent isotype switching from IgG to IgA 99. It was observed that the serum levels of APRIL were elevated in patients with IgAN, and exogenous APRIL could induce more production of Gd-IgA1 in cultured lymphocytes from patients with IgAN 100. Microbial DNA is known to contain oligodeoxynucleotides (ODN) with CpG (CpG-ODN), which mimic immunostimulatory activity. It has been demonstrated that CpG-ODN increased the expression of the transmembrane activator and calcium modulator and cyclophilin ligand interactor on B-cells, enhancing APRIL-induced production of IgA 101. Similarly, BAFF, which is homologous to APRIL, has been shown to exhibit overlapping functions and share receptors with APRIL. Overexpression of BAFF was reported to lead to autoimmune disease with commensal microbe-dependent mesangial IgA deposits in mice 93. In addition, B-cells could directly interact with CD4^+^ T-cells via CD40 in entering the germinal center. In the presence of costimulatory signals, these antigen-stimulated naïve B-cells were demonstrated to undergo physiological proliferation and class switching from IgM to IgA1^+^ plasma cells 102.

Gut microbiota are known to also have a role in enhancing the production of IgA, and, conversely, the produced IgA influences the composition of the microbiota. This complicated relationship between gut microbiota and IgA is critically essential for the host, as dysfunctions in this balance might result in dysbiosis and inflammation in the gut 103. It has been reported that in germ-free mice, little IgA is produced. However, its levels were shown to rapidly increase upon bacterial colonization 104. Alterations in the composition of commensal microbiota, such as increments in *Bacteroides spp*, *Clostridium coccoides*, and *Lactobacillus spp* and reduction in *Bifidobacterium spp*, has been associated with increased levels of secretory IgA in mice 105. Moreover, a recent study has shown that the commensal microbe* Bacteroidales Family S24-7* triggered the propagation and activation of type 2 innate lymphoid cells (ILC2), with the production of IL-5 enhancing B-cell maturation, thus leading to the production of IgA 70. Peyer's patches constitute the most prominent IgA inductive site in the GALT. Another noncultivable bacteria, *Alcaligenes*, has been found to invade the isolated lymphoid follicle and Peyer's patches, driving the production of IgA in both humans and animals 106. Different microbial species might have distinct IgA-inducing potentials, that is, *Bacteroides ovatus* has been demonstrated to strongly stimulate the increased production of IgA in the large intestine of mice through the T cell-dependent B-cell-activation pathway 107. Changes in the expression of the polymeric immunoglobulin receptor, factors affecting the transport of dimeric IgA, or increased formation of IgA^+^ plasma cells in the Peyer's patches have been suggested to be responsible for these differences 108. Besides, continuous stimulation has been considered as not being an absolute requirement for the production of IgA as short-term reversible colonization with *Escherichia coli K-12* could also lead to long-term production of IgA in germ-free mice 24.

Intestinal epithelial cells are known to express different pattern recognition receptors, such as TLRs and NOD-like receptors, for immune surveillance. They have been shown to sense bacterial components, and induce signal transduction, promoting to the proliferation of epithelial cells and the release of cytokines, antimicrobial peptides, and mucus by intestinal epithelial cells. For instance, lipopolysaccharides, the outer membrane of Gram-negative bacteria, has been observed to have a capacity for activating TLR4 in cultured peripheral B-lymphocytes isolated from IgAN. More specifically, LPS was shown to strongly inhibit the expression of the core I beta3-Gal-T-specific molecular chaperone (Cosmc) mRNA, resulting in the overproduction of polymeric Gd-IgA1 109. Flagellin, derived from invasive pathogens, was suggested to induce proinflammatory cytokines by TLR-5+ CD11c^high^ dendritic cells in intestinal lamina propria, resulting in increased IgA in mice 110,111. Uremic toxins, such as indoxyl sulfate, p-cresyl sulfate, indole-3 acetic acid, trimethylamine N-oxide, and phenylacetylglutamine, were also found to promote the release of TNF-α, IFN-γ, IL-1β, IL-12, resulting in intestinal inflammation and the overproduction of IgA 112. Specifically, indoxyl sulfate was observed to induce intestinal barrier injury through IRF1-DRP1 axis-mediated mitophagy impairment. Reducing the concentration of indoxyl sulfate or targeting the IRF1-DRP1 axis might be promising therapeutic strategy to palliate chronic kidney disease-associated intestinal dysfunction 113.

## The synergetic role of diet in amplifying IgA/Gd-IgA1 immunity

Intestinal infections and gut microbiome are both correlated with the host immune status, and especially the production of IgA1. Accordingly, the diet might amplify their effects either directly or indirectly.

A high-fat diet (HFD) has been associated with many chronic complications, including type 2 diabetes, cardiovascular disease, and kidney disease. More specifically, it was shown to induce inflammation, increase intestinal permeability, and alter the microbial composition of the intestine 114. In addition, HFDs has also been shown to reduce the frequency and quantity of IgA-producing plasma cells (IgA^+^B220^-^) in the colonic lamina propria in mice models. Moreover, in a gene expression profiling report, a decrease in the expression of the *Aldh1a1*, *Il5*, *Tgfb1*, and *Tnfsf13* (*APRIL*) was shown 115. This might indicate that HFDs are not entirely detrimental in IgAN, but additional evidence is needed to delineate the potential mechanisms. Gluten has also been related to intestinal immune hyperresponsiveness, potentially driving increased intestinal permeability. Changes in the permeability of the intestine might lead to antigen access to the systemic circulation and increased production of Gd-IgA1 116. It was observed that gluten promoted the production of IgA1 in the intestine, eliciting intestinal inflammation, and increasing the production of antigliadin antibodies. It was further shown that gluten also enhanced the shedding of CD89 from the surface of monocytes/macrophages and induced the formation of IgA1-sCD89 ICs in α1^KI^-CD89^TG^ humanized mice, another IgAN mouse model. Not surprisingly, a gluten-free diet was suggested to be associated with a decrease of mesangial IgA1 deposits and hematuria 117. Based on this, it was assumed that patients with IgAN might also benefit from a gluten-free diet, with reduced levels of IgA-containing ICs, antigliadin IgA in the circulation, as well as reduced proteinuria and microscopic hematuria 118.

Other dietary antigens, such as bovine serum albumin (BSA), and β-lactoglobulin, have also been shown to have a role in promoting the synthesis of IgA-containing ICs. Orally administered proteins are absorbed by the adult mammalian intestine. As such, impairment of the barrier function of the intestinal mucosa or intestinal inflammation might result in increased absorption of these macromolecules. Accordingly, it has been observed that the anti-BSA antibody and levels of ICs were positively correlated, and the BSA antigen was present in IgA-containing ICs in patients with IgAN 119. Oral challenges with milk from cows (β-lactoglobulin) in patients with IgAN were reported to promote the elevated levels of IgA-containing ICs and could be inhibited by the sodium cromoglycate antiallergic agent 120. These findings have provided a view of the dietary antigens in the regulation of intestinal immune responses in the development of IgAN.

## Supporting genetic evidence from genome-wide association studies

Additional evidence from genome-wide association studies (GWASs) has emphasized the importance of intestinal immune responses in IgAN. Recent GWASs in IgAN have identified several susceptibility loci, such as *CARD9*, *HORMAD2*, *ITGAM, VAV,* and *HLA-DQ/HLA-DR*. These genes were suggested to be involved in the maintenance of the intestinal epithelial barrier, the immune response to intestinal pathogens, and the intestinal production of IgA 121. In addition, these loci were shown to be shared among IgAN, IBD, and microbial infections. In particular, CARD9 has been reported to assist intestinal repair by promoting the production of IL-22 or coordinating Th17- and innate lymphoid cell-mediated intestinal immune responses. It has also been observed to have a role in shaping the compositions of bacteria and fungi 122,123. The *ITGAM* gene encodes the α_M_β_2_-integrin α-chain (also known as Mac-1, CR3, and CD11b/CD18). It has been shown that CD11b^+^ IgA^+^ plasma cells produce more IgA compared with CD11b^-^ IgA^+^ plasma cells 124. In addition, VAV3, along with VAV1 and VAV2, are Rho guanine nucleotide exchange factors that are known to be essential for adaptive immune function and have been involved in the development and activation of lymphocytes 125. The VAV proteins have been shown to contribute to the activation of NF-κB in B-cells, which might be necessary for the production of IgA 126. Lastly, the combined polygene risk scores from GWAS were found to be significantly correlated with variations in local pathogens, as evidenced by the concordant geospatial distribution with helminth diversity. From a non-hypothesis way, these data implied that host-intestinal pathogen interactions were involved in shaping IgAN 121.

## Therapeutic strategies with an updated pathophysiological perspective

**Figure 3:**
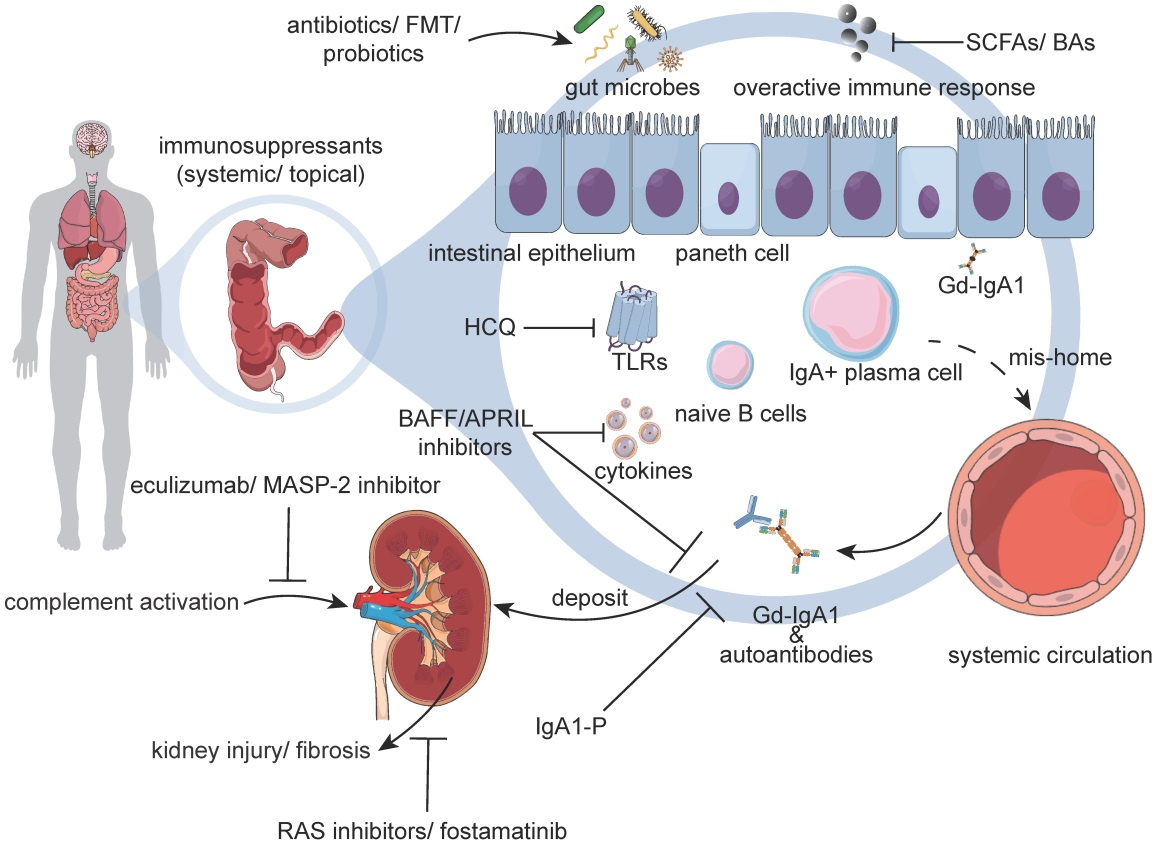
**Potential IgAN Therapies.** Immunosuppressants reduce immune hyperresponsiveness, but their effectiveness needs further verification. Emerging interventions have demonstrated that antibiotics, FMTs, probiotics, and the use of specific metabolites might be effective in treating IgAN. These treatments were shown to restore a diverse community of microbes, relieve mucosal immune responses, and decrease the production of IgA. For downstream interventions, HCQ inhibits excessive TLR activation, and BAFF/APRIL inhibitors restrict the survival and transformation of B-cells. IgA1-protease reduces IgA1 in serum and IgA1 deposits in the mesangium. Besides, eculizumab or the MASP-2 inhibitor strict the activation of the complement system, and RAS inhibitors or fostamatinib prevent kidney injuries and fibrosis. Abbreviations: APRIL: a proliferation-inducing ligand; BAFF: B cell-activating factor; BAs: bile acids; FMTs: fecal microbiota transplantations; Gd-IgA1: galactose-deficient IgA1; HCQ: hydroxychloroquine; IgA1-P: IgA1 protease; MASP-2: mannan-binding lectin-associated serine protease-2; RAS: renin-angiotensin system; SCFAs: short-chain fatty acids; TLRs: toll-like receptors.

Currently, there are no disease-specific therapeutics for IgAN, primarily because of its unclear pathogenesis. It has been hypothesized that the alterations of the gut microbiome and undue IgA immunity in IgAN, might lead to the overproduction of IgA/Gd-IgA1 in genetically susceptible individuals. Thus, in an updated pathophysiological perspective, modulation of the gut microbiota, suppression of the excessive mucosal immune responses, inhibition of the production of pathogenic IgA1/ICs, and dampening of inner-kidney injuries may be promising strategies in the future (**Figure 3**).

### Modulating the gut microbiota

Considering the pathogenesis of IgAN, the process of modulating the gut microbiota would mainly involve the elimination of pathogens, restoration of the microbial diversity, and regulation of metabolites to normal levels. The gut microbiome has been suggested to stimulate or palliate proinflammatory immune responses. This would be driven either by bacteria themselves, or by their produced metabolites, the immune cells they train, or another mechanism. Thus, various routes for potential IgAN therapies against the gut microbiome are under investigation, including nonspecific antibiotics, supplements of specific metabolites, and fecal microbiota transplantation (FMT).

A recent study revealed that in humanized α1^KI^-CD89^Tg^ mice, broad-spectrum antibiotics could decrease the levels of hIgA1-mIgG ICs in the circulatory system. Moreover, they were also shown to prevent the mesangial deposition of hIgA1 and decrease proteinuria 94. However, the administration of antibiotics must be performed with caution due to the risk of indiscriminate use leading to a series of adverse events, such as *Clostridioides difficile* infections and antibiotic resistance. At present, novel approaches have been emerging. Researchers have developed programmed inhibitor cells, which direct the antibacterial activity of the type VI secretion system against specified species or strains of bacteria. This kind of intervention has been suggested to not affect the composition of the normal microbial community, with rare resistance 127. Concomitantly, aberrant changes in microbial metabolites and modulation of the innate immune response of the host are incentives in many autoimmune diseases, metabolic disorders, and intestinal infections 128-130. Among many metabolites, short-chain fatty acids (SCFAs) have been in the spotlight. Bacteria break down partially digested complex carbohydrates via fermentation, producing SCFAs, such as acetate, butyrate, and propionate. Acetate has been shown to decrease the number of IgA^+^ B cells and the proportion of CD8^+^ T-cells in Peyer's patches, while increasing the percentage of Tregs in NOD mice 131. Butyrate is known to stimulate the colonic epithelium to synthesize IL-18 via the activation of the G protein-coupled receptor 109a to lessen inflammation in mice 128. Another potential option is the regulation of intestinal immune responses by bile acids (BAs). Briefly, BAs are synthesized within hepatocytes, released into the duodenum, and are known to increase the quantity of colonic RORγ^+^ Tregs, ameliorating the susceptibility of the host to inflammatory colitis in mice 132. Thus, SCFAs and BAs might decrease the production of IgA and palliate overactive immune responses.

Apart from the use of antibiotics and specific metabolites, several novel therapies for immune conditions, such as FMT or the use of probiotics/prebiotics are being developed. More specifically, FMT was found to combat the cyclical diseased state by restoring a diverse community of microbes 133. Besides, in patients with ulcerative colitis, FMT could also increase the levels of the biosynthesis of SCFAs and secondary BAs 134. Mitigating immune responses or increasing the concentrations of SCFAs and BAs by FMT might decrease the production of IgA. Clinical trials about the safety and efficacy of FMT in patients with IgAN are currently being conducted (NCT03633864). Meanwhile, the past decade has witnessed the rapidly accumulated research on probiotics or prebiotics as a means to modulate the gut microbiota and host immune responses 135. Probiotic strains, just like lactic acid bacteria, have been suggested to increase the levels of anti-inflammatory cytokines, such as IL-10 *in vivo* colitis models 136. Some *Lactobacillus* and *Bifidobacterium* genera were also reported to produce lactic and acetic acids to lower the luminal pH and decrease the production of IgA in mice 131,137.

### Suppressing excessive mucosal immune responses

Intestinal immune disorders contribute to the overproduction of Gd-IgA1 and O-glycan-specific antibodies. Suppressing the excessive mucosal immune responses seems to be a promising therapeutic approach. Accordingly, the 2012 KGIDO guidelines suggested that patients with persistent proteinuria ≥1 g/d, despite 3-6 mo of optimized supportive care and GFR > 50 mL/min per 1.73 m^2^, should receive a 6 mo course of corticosteroid therapy. Based on this, these guidelines have also suggested the use of steroids in patients with IgAN and rapidly progressive crescentic IgAN 138. In the “STOP” trial, some of the patients with IgAN receiving supportive care plus immunosuppressive therapy were reported to have a full clinical remission as defined by the changes in proteinuria measured with a protein-to-creatinine ratio, and renal function. However, supportive care plus immunosuppressive treatment could not improve their outcome 139. In the “TESTING” trial, the decline of the annual rate of estimated glomerular filtration rate, or the time-averaged proteinuria in patients with IgAN was shown to be lower in the methylprednisolone group 140. Systemic immunosuppressants, such as budesonide, are subjected to high first-pass metabolism, resulting in low systemic exposure (about 10% of the administered dose). The use of these systemic immunosuppressants, however, would cause adverse effects, such as severe infections, hyperglycemia, or intestinal perforation. Thus, the clinical benefit of the use of corticosteroids in IgAN remains unestablished. Nonetheless, treatment of patients using prednisone plus cyclophosphamide or mycophenolate mofetil in combination with corticosteroid has been in progress in patients with advanced-stage IgAN (NCT03218852; NCT02981212). Some novel therapeutic strategies, such as using the macrophage-derived microvesicle for kidney-targeted delivery of dexamethasone, had been shown to suppress renal inflammation and fibrosis without apparent glucocorticoid adverse effects 141.

Considering that the number of IgA^+^ plasma cells and levels of total IgA have been shown to be lower in the large than in the small intestine, intestinal immune responses in the small intestine require particular attention 142. To this end, the ileum targeting-release immunosuppressants, where the Peyer's patches are enriched, represent an emerging class of medicines designed to control the local immune hyperresponsiveness with minimal systemic adverse effects among patients with IgAN. In a phase 2b trial, NEFECON was reported to decrease the levels of proteinuria, and appeared to be safe and well-tolerated 62. In the future, larger trials of longer duration will be needed to quantify the magnitude of relative risk reduction associated with this kind of therapy at risk of progression to end-stage renal disease. Besides, similar studies should be conducted in other populations. According to the pharmacokinetic data about Entocort™ EC, systemic exposure of budesonide (9 mg) was reported to be 9.6 ± 3.6%, and the maximum concentration was 4.9 ± 0.8 μg/L 143. Increased systemic exposure of budesonide has been observed in patients with IBD compared with healthy controls 143. However, there are no pharmacokinetic data on NEFECON in patients with IgAN. Since a part of NEFECON is known to be absorbed, there are some concerns about its systemic effect in patients with IgAN, which might directly affect mucosal immune responses in NALT. Further studies are needed to clarify its systemic effects. Meanwhile, a phase 3 multicenter study aiming to evaluate the efficacy, safety, and tolerability of the administration of oral NEFECON in patients with primary IgAN on a background of optimized RAS inhibitor therapy is currently underway (NCT03643965).

### Inhibiting the Production of Pathogenic IgA1/IgA1-ICs

The class switch of B-cells involves both T-cell-dependent and T-cell-independent pathways. During this process, naïve B-cells transform into IgA^+^ plasma cells, secreting Gd-IgA1 to the apical surface. The T-cell-dependent activation pathway involves the activation of inflammatory cells, autoantigen presentation, expression of TLRs, as well as the production of cytokines or chemokines. Thus, pharmacological inhibition of the above pathways seems a feasible strategy to ameliorate immune hyperresponsiveness. More recently, a number of potent immunomodulatory drugs, such as hydroxylchloroquine (HCQ), have been introduced in the clinical setting. It was shown that HCQ inhibited the excessive activation of the immune system signaled by TLR9 both *in vivo* and *in vitro* 144,145, and significantly decreased the level of IgA and proteinuria in patients with IgAN and rats 146,147. Regarding the T-cell independent mechanism, atacicept, a dual BAFF/APRIL inhibitor, was shown to be both effective and tolerated. More specifically, atacicept decreased the serum levels of IgA in patients with systemic lupus erythematosus by 45% - 50% 148. A phase 2 clinical trial to test its safety, tolerability, and dose-response in patients with IgAN has just been completed (NCT02808429). Similarly, anti-APRIL antibody treatment in grouped ddy mice, a spontaneous IgAN model, was demonstrated to significantly decrease albuminuria, serum levels of IgA, and deposition of glomerular IgA 149. Likewise, VIS649, the humanized IgG2κ antibody targeting and neutralizing human APRIL through unique epitope engagement, was also reported to decrease the serum levels of IgA by up to 70% in non-human primates 150. BION-1301, a first-in-class humanized IgG4 anti-APRIL monoclonal antibody, is currently under investigation in a phase 1 trial among adults with IgAN (NCT03945318).

B-cell depletion therapy aims to control the autoimmune responses in patients with autoimmune diseases. Rituximab, the monoclonal antibody directed against the CD20 antigen, has been demonstrated to effectively deplete B-cells. Accordingly, B-cell depletion therapy has been shown to be effective in patients with rheumatoid arthritis or primary membranous nephropathy 151,152. Thus, from the perspective of disease pathogenesis, this kind of treatment might also be effective in patients with IgAN. However, from the perspective of the clinical trial, it was reported that it could not reduce the level of proteinuria, Gd-IgA1, or auto-antibodies in patients with IgAN 153. The authors put forward a few scenarios accounting for the ineffectiveness. For instance, rituximab could not effectively deplete long-lived plasma cells. These plasma cells are known to be derived from B-cells, but in the process, they have lost the expression of CD19. Unaffected plasma cells have been shown to still produce antiglycan antibodies, with IgAN displaying a prominent involvement in the mucosal immune responses. Therefore, B-cell depletion therapy might not provide long-lasting effects. Bortezomib is a proteasome inhibitor known to target plasma cells. Short term use of bortezomib in select cases of IgAN has demonstrated promising ramifications in reducing proteinuria 154. A similar unexpected efficiency of rituximab could also be observed in patients with lupus nephritis 155. Obinutuzumab, the humanized anti-CD20 IgG1 type 2 monoclonal antibody, was shown to partially overlap with the epitope recognized by rituximab 156. Accordingly, more recent reports comparing obinutuzumab to rituximab have suggested that longer-term effective B-cell depletion would show increased efficiency in disease remission in lupus 157. As IgAN is a complex disease, different pathways might play different roles in disease pathogenesis. Thus, a protocol of precision medicines should be the main focus in the future. The potential effects of rituximab in decreasing the circulating polymeric forms of IgA and slowing down disease progression of patients with recurrent IgAN are being currently tested (NCT02571842).

The apoptosis inhibitor of macrophage (AIM) is a circulating protein and a member of the scavenger receptor cysteine-rich superfamily. It is known to be exclusively secreted by tissue macrophages, which might also drive the phlogogenic cascade 158. In particular, AIM is known to modulate the phagocytotic function of macrophages, supporting their survival 159. As AIM has the capacity to bind to LPS from gram-negative bacteria, it was suggested that it might act as a pattern recognition molecule 160. In IgAN, the expression of AIM could be detected in the glomeruli, tubular epithelial cells, and infiltrating macrophages in the glomeruli and interstitial area. The ratio of levels of AIM/creatinine was found to be significantly correlated with proteinuria and hematuria. The urinary levels of AIM were demonstrated to function as a biomarker for disease activity among patients with IgAN and Henoch-Schönlein purpura nephritis 161. In addition, it was demonstrated that AIM was required for the overall inflammatory glomerular injury following IgA deposition in grouped ddY mice. Based on this, it was assumed that AIM might play a significant role in the formation of ICs, facilitating disease progression 162. Therefore, blockage of AIM deposition presents to be a novel target in the treatments of IgAN.

Regarding the already existing IgA1, the IgA1 protease (IgA1-P), which has been reported to function in aiding clearance has provided new insights toward therapeutic strategies for IgAN. Several pathogenic microbes (e.g., *Sutterella*, *Streptococcus pneumonia*, *Haemophilus influenza*, and *Neisseria meningitides)* were shown to degrade IgA by secreting IgA1-P 163, which cleaves the hinge region of human IgA1 164,165. The recombinant IgA1-P has been shown to markedly reduce IgA1 in the serum and IgA1 deposits in the mesangium, and mitigate inflammation, fibrosis, and hematuria in the α1^KI^-CD89^Tg^ mouse model of IgAN 166. This kind of treatment seems to represent a potential option for a future specific therapy of IgAN. The IgA receptor family consists of the CD89 polymeric immunoglobulin receptor, the Fcα/μR, and two other alternative IgA receptors 167. The CD89 protein is known to be expressed on Kupffer cells of the liver, neutrophils, eosinophils, monocytes/macrophages, and dendritic cells. Interestingly, IgA was shown to be eliminated from the circulation via a CD89-mediated process 168,169. Hosts with hepatobiliary dysfunction, such as liver cirrhosis and bile duct ligation, have been shown to exhibit elevated levels of IgA in their sera, mesangial proliferation in kidneys, and hematuria 170,171. Thus, enhancing the functions of IgA receptors in clearing the excess IgA1 or ICs might hold great promise.

### Dampen inner-kidney injury

Several current therapeutic approaches have aimed in preventing subsequent damage to the kidneys, including the use of inhibitors of the complement system, RAS, and antifibrotic agents.

The classical complement pathway is known to typically require ICs for activation. However, evidence of the activation of the classical pathway, such as C1q deposits, has been lacking in kidney biopsy specimens of patients with IgAN. Instead, IgAN is also characterized by deposits of C3, properdin, C4d, mannose-binding lectin, and C5b-9 11. Thus, the complement system has been regarded to be activated via the mannose-binding lectin and alternative pathway, leading to glomerular injury and tubulointerstitial scarring 38. Therefore, blocking the cascade activity of the complement system might be an essential target in further improving outcomes. Eculizumab is a known inhibitor of the terminal complement pathway that binds C5, preventing the formation of the membrane attack complexes of complement. In a case report, it was shown that early initiation of eculizumab therapy in a patient with progressive IgAN was able to reduce proteinuria and stabilize the glomerular filtration rate 172. However, clinically adequate proof to validate its effectiveness is still lacking. Another therapeutic targeting the C5 component of the complement pathway is cemdisiran, which is being currently evaluated on its effect on proteinuria in adults with IgAN (NCT03841448). Mannose-binding lectins are known to induce the activation of the complement cascade via 2 types of mannan-binding lectin-associated serine protease-1/2 (MASP-1/2). Thus, MASP-2, the effector enzyme of the lectin pathway, could provide novel perspectives for the treatment of the overactive complement system. Narsoplimab is a human monoclonal antibody targeting MASP-2, and related clinical trials of its effectiveness are under active investigation in patients with IgAN (NCT03608033; NCT02682407). Moreover, APL-2, an investigational drug, has been designed to inhibit the complement cascade by centrally targeting at C3. Therefore, it might have the potential to treat a wide range of complement-mediated diseases more effectively than is currently possible using partial inhibitors of complement, such as in patients with IgAN, lupus nephritis, or C3 glomerulonephritis (NCT03453619). Other complement-based therapies include the anticomplement factor B monoclonal inhibitor (IONIS-FB-LRx) and plasma therapy, which are all under active investigation in patients with primary or severe crescentic IgAN (NCT04014335; NCT02647255).

RAS inhibitors are the current mainstream treatment options in IgAN due to their efficacy in reducing proteinuria and slowing down disease progression 173. The application of RAS inhibitors has shown a significant beneficial effect on patients with histologically advanced IgAN with tubuleinterstitial lesions. Multivariate Cox regression analysis showed that the RAS inhibitor was an independent factor in slowing down disease progression 174. Aliskiren is the first in a class of drugs called direct renin inhibitors and has been demonstrated to reduce the mean ratio of urinary protein-to-creatinine, the activity of renin in plasma, and the level of IL-6 or transforming growth factor-β in patients with IgAN 175. However, its effects on renal function and progress of renal disease in hypertensive patients with IgAN need further verification (NCT01184599). In addition, sparsentan, a novel small-molecule candidate, is a dual-acting angiotensin receptor blocker and endothelin receptor antagonist. Respectively, its potential of nephronprotective treatment is being tested in patients with IgAN (NCT03762850).

In IgAN, disease progression can ultimately lead to fibrosis. Current trials are attempting to address the use of the spleen tyrosine kinase (SYK) as an early intermediate in intracellular signal transduction cascades for the B-cell and immunoglobulin Fc receptors. Therefore, SKY seems to be necessary for the proliferation, differentiation, and activation of B-cells 176. The introduction of fostamatinib, an SYK inhibitor, in an autoimmune model of renal disease, showed that it could lead to cessation of autoantibody production and reversal of renal injury 177. Besides, mice treated with fostamatinib were also reported to display less fibrosis and inflammation 178. It is worth looking forward to the results from a global, randomized, double-blind, placebo-controlled trial investigating the safety and efficacy of fostamatinib in patients with IgAN (NCT02112838). The increased expression of the growth arrest-specific 6 (GAS6), which has been strongly implicated in disease progression and mortality, has been demonstrated in endothelial, mesangial cells, and podocytes in IgAN 179. Moreover, AVB-S6-500 was found to suppress fibrosis by capturing circulating and bound GAS6. Thus, a phase 2a clinical study designed to evaluate the safety and efficacy of AVB-S6-500 in patients with IgAN is currently underway (NCT04042623).

## Concluding remarks and prospects

This review integrates existing evidence and highlights the role of aberrant mucosal immune responses in the pathogenesis of IgAN from the perspectives of infections and the gut microbiome. As more in-depth insights into the relevant mechanisms become available, innovative therapeutic strategies to improve IgA-immunity related diseases would be available.

## Abbreviations

APRIL: a proliferation-inducing ligand
AIM: apoptosis inhibitor of macrophage protein
BAFF: B-cell-activating factor
BAs: bile acids
BSA: bovine serum albumin
FMT: fecal microbiota transplantation
GALT: gut-associated lymphoid follicles
Gd-IgA1: galactose-deficient IgA1
GWASs: genome-wide association studies
HFD: high-fat diet
HCQ: hydroxychloroquine
IgA1-P: IgA1 protease
IgAN: IgA nephropathy
IBD: inflammatory bowel disease
ICs: immune complexes
IgA: immunoglobulin A
IL: interleukin
IFN: interferon
MALT: mucosa-associated lymphoid tissue
NALT: nasopharynx-associated lymphoid tissue
NF-κB: nuclear factor-kappa B
RAS: renin-angiotensin system
SCFAs: short-chain fatty acids
SYK: spleen tyrosine kinase
TLRs: Toll-like receptors
Tregs: regulatory T cells

## References

[1] Schena FP, Nistor I (2018). Epidemiology of IgA Nephropathy: A Global Perspective. Semin Nephrol.

[2] D'Amico G (2004). Natural history of idiopathic IgA nephropathy and factors predictive of disease outcome. Semin Nephrol.

[3] Hastings MC, Moldoveanu Z, Julian BA, Novak J, Sanders JT, McGlothan KR (2010). Galactose-deficient IgA1 in African Americans with IgA nephropathy: serum levels and heritability. Clin J Am Soc Nephrol.

[4] Moldoveanu Z, Wyatt RJ, Lee JY, Tomana M, Julian BA, Mestecky J (2007). Patients with IgA nephropathy have increased serum galactose-deficient IgA1 levels. Kidney Int.

[5] Berthoux F, Suzuki H, Thibaudin L, Yanagawa H, Maillard N, Mariat C (2012). Autoantibodies targeting galactose-deficient IgA1 associate with progression of IgA nephropathy. J Am Soc Nephrol.

[6] Chen P, Yu G, Zhang X, Xie X, Wang J, Shi S (2019). Plasma Galactose-Deficient IgA1 and C3 and CKD Progression in IgA Nephropathy. Clin J Am Soc Nephrol.

[7] Zhao N, Hou P, Lv J, Moldoveanu Z, Li Y, Kiryluk K (2012). The level of galactose-deficient IgA1 in the sera of patients with IgA nephropathy is associated with disease progression. Kidney Int.

[8] Zhao YF, Zhu L, Liu LJ, Shi SF, Lv JC, Zhang H (2017). Pathogenic role of glycan-specific IgG antibodies in IgA nephropathy. BMC Nephrol.

[9] Suzuki H, Kiryluk K, Novak J, Moldoveanu Z, Herr AB, Renfrow MB (2011). The pathophysiology of IgA nephropathy. J Am Soc Nephrol.

[10] Yeo SC, Cheung CK, Barratt J (2018). New insights into the pathogenesis of IgA nephropathy. Pediatr Nephrol.

[11] Maillard N, Wyatt RJ, Julian BA, Kiryluk K, Gharavi A, Fremeaux-Bacchi V (2015). Current Understanding of the Role of Complement in IgA Nephropathy. J Am Soc Nephrol.

[12] Lai KN, Tang SC, Schena FP, Novak J, Tomino Y, Fogo AB (2016). IgA nephropathy. Nat Rev Dis Primers.

[13] Floege J, Feehally J (2016). The mucosa-kidney axis in IgA nephropathy. Nat Rev Nephrol.

[14] Papista C, Berthelot L, Monteiro RC (2011). Dysfunctions of the Iga system: a common link between intestinal and renal diseases. Cell Mol Immunol.

[15] Perse M, Veceric-Haler Z (2019). The Role of IgA in the Pathogenesis of IgA Nephropathy. Int J Mol Sci.

[16] Novak J, Julian BA, Tomana M, Mestecky J (2008). IgA glycosylation and IgA immune complexes in the pathogenesis of IgA nephropathy. Semin Nephrol.

[17] Bunker JJ, Erickson SA, Flynn TM, Henry C, Koval JC, Meisel M (2017). Natural polyreactive IgA antibodies coat the intestinal microbiota. Science.

[18] Duerkop BA, Vaishnava S, Hooper LV (2009). Immune Responses to the Microbiota at the Intestinal Mucosal Surface. Immunity.

[19] Diamond G, Beckloff N, Weinberg A, Kisich KO (2009). The roles of antimicrobial peptides in innate host defense. Curr Pharm Des.

[20] Yel L (2010). Selective IgA deficiency. J Clin Immunol.

[21] Macpherson AJ, Yilmaz B, Limenitakis JP, Ganal-Vonarburg SC (2018). IgA Function in Relation to the Intestinal Microbiota. Annu Rev Immunol.

[22] Bunker JJ, Bendelac A (2018). IgA Responses to Microbiota. Immunity.

[23] Rollino C, Vischini G, Coppo R (2016). IgA nephropathy and infections. J Nephrol.

[24] Hapfelmeier S, Lawson MA, Slack E, Kirundi JK, Stoel M, Heikenwalder M (2010). Reversible microbial colonization of germ-free mice reveals the dynamics of IgA immune responses. Science.

[25] Allen AC, Feehally J (2000). IgA1 glycosylation and the pathogenesis of IgA nephropathy. Am J Kidney Dis.

[26] Turnbaugh PJ, Ley RE, Hamady M, Fraser-Liggett CM, Knight R, Gordon JI (2007). The Human Microbiome Project. Nature.

[27] De Angelis M, Montemurno E, Piccolo M, Vannini L, Lauriero G, Maranzano V (2014). Microbiota and metabolome associated with immunoglobulin A nephropathy (IgAN). PLoS One.

[28] Hu X, Du J, Xie Y, Huang Q, Xiao Y, Chen J (2020). Fecal microbiota characteristics of Chinese patients with primary IgA nephropathy: a cross-sectional study. BMC Nephrol.

[29] Alley CD (1987). Human bone marrow-derived IgA is produced by IgA-committed B cells *in vitro*. J Clin Immunol.

[30] Hiki Y, Odani H, Takahashi M, Yasuda Y, Nishimoto A, Iwase H (2001). Mass spectrometry proves under-O-glycosylation of glomerular IgA1 in IgA nephropathy. Kidney Int.

[31] Allen AC, Bailey EM, Brenchley PE, Buck KS, Barratt J, Feehally J (2001). Mesangial IgA1 in IgA nephropathy exhibits aberrant O-glycosylation: observations in three patients. Kidney Int.

[32] Wehbi B, Oblet C, Boyer F, Huard A, Druilhe A, Paraf F (2019). Mesangial Deposition Can Strongly Involve Innate-Like IgA Molecules Lacking Affinity Maturation. J Am Soc Nephrol.

[33] Bene MC, Faure G, Duheille J (1982). IgA nephropathy: characterization of the polymeric nature of mesangial deposits by *in vitro* binding of free secretory component. Clin Exp Immunol.

[34] Saha MK, Julian BA, Novak J, Rizk DV (2018). Secondary IgA nephropathy. Kidney Int.

[35] Scicchitano R, Husband AJ, Clancy RL (1984). Contribution of intraperitoneal immunization to the local immune response in the respiratory tract of sheep. Immunology.

[36] Tuma P, Hubbard AL (2003). Transcytosis: crossing cellular barriers. Physiol Rev.

[37] Boyd JK, Cheung CK, Molyneux K, Feehally J, Barratt J (2012). An update on the pathogenesis and treatment of IgA nephropathy. Kidney Int.

[38] Floege J, Barbour SJ, Cattran DC, Hogan JJ, Nachman PH, Tang SCW (2019). Management and treatment of glomerular diseases (part 1): conclusions from a Kidney Disease: Improving Global Outcomes (KDIGO) Controversies Conference. Kidney Int.

[39] Buren M, Yamashita M, Suzuki Y, Tomino Y, Emancipator SN (2007). Altered expression of lymphocyte homing chemokines in the pathogenesis of IgA nephropathy. Contrib Nephrol.

[40] Kennel-De March A, Bene MC, Hurault de Ligny B, Kessler M, Faure GC (1997). Enhanced expression of CD31 and CD54 on tonsillar high endothelial venules in IgA nephropathy. Clin Immunol Immunopathol.

[41] Feehally J, Beattie TJ, Brenchley PE, Coupes BM, Mallick NP, Postlethwaite RJ (1986). Sequential study of the IgA system in relapsing IgA nephropathy. Kidney Int.

[42] Suzuki Y, Suzuki H, Nakata J, Sato D, Kajiyama T, Watanabe T (2011). Pathological role of tonsillar B cells in IgA nephropathy. Clin Dev Immunol.

[43] Suzuki Y, Suzuki H, Sato D, Kajiyama T, Okazaki K, Hashimoto A (2011). Reevaluation of the mucosa-bone marrow axis in IgA nephropathy with animal models. Adv Otorhinolaryngol.

[44] Inoue T, Sugiyama H, Kitagawa M, Takiue K, Morinaga H, Kikumoto Y (2011). Abnormalities of glycogenes in tonsillar lymphocytes in IgA nephropathy. Adv Otorhinolaryngol.

[45] Nakata J, Suzuki Y, Suzuki H, Sato D, Kano T, Yanagawa H (2014). Changes in nephritogenic serum galactose-deficient IgA1 in IgA nephropathy following tonsillectomy and steroid therapy. PLoS One.

[46] Horie A, Hiki Y, Odani H, Yasuda Y, Takahashi M, Kato M (2003). IgA1 molecules produced by tonsillar lymphocytes are under-O-glycosylated in IgA nephropathy. Am J Kidney Dis.

[47] Bene MC, Faure G, Hurault de Ligny B, Kessler M, Duheille J (1983). Immunoglobulin A nephropathy. Quantitative immunohistomorphometry of the tonsillar plasma cells evidences an inversion of the immunoglobulin A versus immunoglobulin G secreting cell balance. J Clin Invest.

[48] Bene MC, Hurault De Ligny B, Kessler M, Faure GC (1991). Confirmation of tonsillar anomalies in IgA nephropathy: a multicenter study. Nephron.

[49] Khodadadi L, Cheng Q, Radbruch A, Hiepe F (2019). The Maintenance of Memory Plasma Cells. Front Immunol.

[50] Fujieda S, Suzuki S, Sunaga H, Yamamoto H, Seki M, Sugimoto H (2000). Induction of IgA against Haemophilus parainfluenzae antigens in tonsillar mononuclear cells from patients with IgA nephropathy. Clin Immunol.

[51] Watanabe H, Goto S, Mori H, Higashi K, Hosomichi K, Aizawa N (2017). Comprehensive microbiome analysis of tonsillar crypts in IgA nephropathy. Nephrol Dial Transplant.

[52] Feenstra B, Bager P, Liu X, Hjalgrim H, Nohr EA, Hougaard DM (2017). Genome-wide association study identifies variants in HORMAD2 associated with tonsillectomy. J Med Genet.

[53] D'Amelio R, Palmisano L, Le Moli S, Seminara R, Aiuti F (1982). Serum and salivary IgA levels in normal subjects: comparison between tonsillectomized and non-tonsillectomized subjects. Int Arch Allergy Appl Immunol.

[54] Bock A, Popp W, Herkner KR (1994). Tonsillectomy and the immune system: a long-term follow up comparison between tonsillectomized and non-tonsillectomized children. Eur Arch Otorhinolaryngol.

[55] Jung KY, Lim HH, Choi G, Choi JO (1996). Age-related changes of IgA immunocytes and serum and salivary IgA after tonsillectomy. Acta Otolaryngol Suppl.

[56] Xie Y, Chen X, Nishi S, Narita I, Gejyo F (2004). Relationship between tonsils and IgA nephropathy as well as indications of tonsillectomy. Kidney Int.

[57] Ikinciogullari A, Dogu F, ikinciogullari A, Egin Y, Babacan E (2002). Is immune system influenced by adenotonsillectomy in children?. Int J Pediatr Otorhinolaryngol.

[58] Wyatt RJ, Julian BA (2013). IgA nephropathy. N Engl J Med.

[59] Wang M, Lv J, Zhang X, Chen P, Zhao M, Zhang H (2020). Secondary IgA Nephropathy Shares the Same Immune Features With Primary IgA Nephropathy. Kidney Int Rep.

[60] MacDermott RP, Schloemann SR, Bertovich MJ, Nash GS, Peters M, Stenson WF (1989). Inhibition of antibody secretion by 5-aminosalicylic acid. Gastroenterology.

[61] Trollmo C, Gudmundsson S, Feltelius N, Rogberg S, Smedegard G, Klareskog L (2007). Sulphasalazine inhibits human antigen-specific immune responses *in vivo*. Ann Rheum Dis.

[62] Fellstrom BC, Barratt J, Cook H, Coppo R, Feehally J, de Fijter JW (2017). Targeted-release budesonide versus placebo in patients with IgA nephropathy (NEFIGAN): a double-blind, randomised, placebo-controlled phase 2b trial. Lancet.

[63] Habura I, Fiedorowicz K, Wozniak A, Idasiak-Piechocka I, Kosikowski P, Oko A (2019). IgA nephropathy associated with coeliac disease. Cent Eur J Immunol.

[64] Park JS, Song JH, Yang WS, Kim SB, Kim YK, Hong CD (1994). Cytomegalovirus is not specifically associated with immunoglobulin A nephropathy. J Am Soc Nephrol.

[65] Tomino Y, Yagame M, Omata F, Nomoto Y, Sakai H (1987). A case of IgA nephropathy associated with adeno- and herpes simplex viruses. Nephron.

[66] Suzuki S, Nakatomi Y, Sato H, Tsukada H, Arakawa M (1994). Haemophilus parainfluenzae antigen and antibody in renal biopsy samples and serum of patients with IgA nephropathy. Lancet.

[67] Sharmin S, Shimizu Y, Hagiwara M, Hirayama K, Koyama A (2004). Staphylococcus aureus antigens induce IgA-type glomerulonephritis in Balb/c mice. J Nephrol.

[68] Iwama H, Horikoshi S, Shirato I, Tomino Y (1998). Epstein-Barr virus detection in kidney biopsy specimens correlates with glomerular mesangial injury. Am J Kidney Dis.

[69] Liu XZ, Zhang YM, Jia NY, Zhang H (2020). Helicobacter pylori infection is associated with elevated galactose-deficient IgA1 in IgA nephropathy. Ren Fail.

[70] Satoh-Takayama N, Kato T, Motomura Y, Kageyama T, Taguchi-Atarashi N, Kinoshita-Daitoku R (2020). Bacteria-Induced Group 2 Innate Lymphoid Cells in the Stomach Provide Immune Protection through Induction of IgA. Immunity.

[71] Yang M, Li FG, Xie XS, Wang SQ, Fan JM (2014). CagA, a major virulence factor of Helicobacter pylori, promotes the production and underglycosylation of IgA1 in DAKIKI cells. Biochem Biophys Res Commun.

[72] Soylu A, Berktas S, Sarioglu S, Erbil G, Yilmaz O, Demir BK (2008). Saccharomyces boulardii prevents oral-poliovirus vaccine-induced IgA nephropathy in mice. Pediatr Nephrol.

[73] Kobayashi N, Bagheri N, Nedrud JG, Strieter RM, Tomino Y, Lamm ME (2003). Differential effects of Sendai virus infection on mediator synthesis by mesangial cells from two mouse strains. Kidney Int.

[74] Hu X, Feng J, Zhou Q, Luo L, Meng T, Zhong Y (2019). Respiratory Syncytial Virus Exacerbates Kidney Damages in IgA Nephropathy Mice via the C5a-C5aR1 Axis Orchestrating Th17 Cell Responses. Front Cell Infect Microbiol.

[75] Miura N, Imai H, Kikuchi S, Hayashi S, Endoh M, Kawamura T (2009). Tonsillectomy and steroid pulse (TSP) therapy for patients with IgA nephropathy: a nationwide survey of TSP therapy in Japan and an analysis of the predictive factors for resistance to TSP therapy. Clin Exp Nephrol.

[76] Muto M, Manfroi B, Suzuki H, Joh K, Nagai M, Wakai S (2017). Toll-Like Receptor 9 Stimulation Induces Aberrant Expression of a Proliferation-Inducing Ligand by Tonsillar Germinal Center B Cells in IgA Nephropathy. J Am Soc Nephrol.

[77] Kawamura T, Yoshimura M, Miyazaki Y, Okamoto H, Kimura K, Hirano K (2014). A multicenter randomized controlled trial of tonsillectomy combined with steroid pulse therapy in patients with immunoglobulin A nephropathy. Nephrol Dial Transplant.

[78] Komatsu H, Fujimoto S, Hara S, Sato Y, Yamada K, Kitamura K (2008). Effect of tonsillectomy plus steroid pulse therapy on clinical remission of IgA nephropathy: a controlled study. Clin J Am Soc Nephrol.

[79] Xie Y, Nishi S, Ueno M, Imai N, Sakatsume M, Narita I (2003). The efficacy of tonsillectomy on long-term renal survival in patients with IgA nephropathy. Kidney Int.

[80] Maruyama S, Gohda T, Suzuki Y, Suzuki H, Sonoda Y, Ichikawa S (2016). Beneficial effects of tonsillectomy plus steroid pulse therapy on inflammatory and tubular markers in patients with IgA nephropathy. Kidney Res Clin Pract.

[81] Rasche FM, Schwarz A, Keller F (1999). Tonsillectomy does not prevent a progressive course in IgA nephropathy. Clin Nephrol.

[82] Piccoli A, Codognotto M, Tabbi MG, Favaro E, Rossi B (2010). Influence of tonsillectomy on the progression of mesangioproliferative glomerulonephritis. Nephrol Dial Transplant.

[83] Vergano L, Loiacono E, Albera R, Coppo R, Camilla R, Peruzzi L (2015). Can tonsillectomy modify the innate and adaptive immunity pathways involved in IgA nephropathy?. J Nephrol.

[84] Feehally J, Coppo R, Troyanov S, Bellur SS, Cattran D, Cook T (2016). Tonsillectomy in a European Cohort of 1,147 Patients with IgA Nephropathy. Nephron.

[85] Brown EM, Kenny DJ, Xavier RJ (2019). Gut Microbiota Regulation of T Cells During Inflammation and Autoimmunity. Annu Rev Immunol.

[86] Kiryluk K, Novak J (2014). The genetics and immunobiology of IgA nephropathy. J Clin Invest.

[87] Stecher B, Maier L, Hardt WD (2013). 'Blooming' in the gut: how dysbiosis might contribute to pathogen evolution. Nat Rev Microbiol.

[88] Coppo R (2015). The intestine-renal connection in IgA nephropathy. Nephrol Dial Transplant.

[89] Atarashi K, Suda W, Luo C, Kawaguchi T, Motoo I, Narushima S (2017). Ectopic colonization of oral bacteria in the intestine drives TH1 cell induction and inflammation. Science.

[90] Chen K, Goodwin M, McAleer J, Nguyen N, Way E, Kolls J (2014). Recombinant outer membrane protein: a potential candidate for Th17 based vaccine against <em>Klebsiella pneumoniae</em>. (VAC7P.967). The Journal of Immunology.

[91] Zielinski CE, Mele F, Aschenbrenner D, Jarrossay D, Ronchi F, Gattorno M (2012). Pathogen-induced human TH17 cells produce IFN-gamma or IL-10 and are regulated by IL-1beta. Nature.

[92] Mayer FL, Wilson D, Hube B (2013). Candida albicans pathogenicity mechanisms. Virulence.

[93] McCarthy DD, Kujawa J, Wilson C, Papandile A, Poreci U, Porfilio EA (2011). Mice overexpressing BAFF develop a commensal flora-dependent, IgA-associated nephropathy. J Clin Invest.

[94] Chemouny JM, Gleeson PJ, Abbad L, Lauriero G, Boedec E, Le Roux K (2018). Modulation of the microbiota by oral antibiotics treats immunoglobulin A nephropathy in humanized mice. Nephrol Dial Transplant.

[95] Wu IW, Gao SS, Chou HC, Yang HY, Chang LC, Kuo YL (2020). Integrative metagenomic and metabolomic analyses reveal severity-specific signatures of gut microbiota in chronic kidney disease. Theranostics.

[96] Wang YF, Zheng LJ, Liu Y, Ye YB, Luo S, Lu GM (2019). The gut microbiota-inflammation-brain axis in end-stage renal disease: perspectives from default mode network. Theranostics.

[97] Kayama H, Okumura R, Takeda K (2020). Interaction Between the Microbiota, Epithelia, and Immune Cells in the Intestine. Annu Rev Immunol.

[98] Nakawesi J, This S, Hutter J, Boucard-Jourdin M, Barateau V, Muleta KG (2020). alphavbeta8 integrin-expression by BATF3-dependent dendritic cells facilitates early IgA responses to Rotavirus. Mucosal Immunol.

[99] Litinskiy MB, Nardelli B, Hilbert DM, He B, Schaffer A, Casali P (2002). DCs induce CD40-independent immunoglobulin class switching through BLyS and APRIL. Nat Immunol.

[100] Zhai YL, Zhu L, Shi SF, Liu LJ, Lv JC, Zhang H (2016). Increased APRIL Expression Induces IgA1 Aberrant Glycosylation in IgA Nephropathy. Medicine (Baltimore).

[101] Takahara M, Nagato T, Nozaki Y, Kumai T, Katada A, Hayashi T (2019). A proliferation-inducing ligand (APRIL) induced hyper-production of IgA from tonsillar mononuclear cells in patients with IgA nephropathy. Cell Immunol.

[102] Pabst O, Slack E (2020). IgA and the intestinal microbiota: the importance of being specific. Mucosal Immunol.

[103] Macpherson AJ, Koller Y, McCoy KD (2015). The bilateral responsiveness between intestinal microbes and IgA. Trends Immunol.

[104] Bos NA, Kimura H, Meeuwsen CG, De Visser H, Hazenberg MP, Wostmann BS (1989). Serum immunoglobulin levels and naturally occurring antibodies against carbohydrate antigens in germ-free BALB/c mice fed chemically defined ultrafiltered diet. Eur J Immunol.

[105] Aguilera M, Cerda-Cuellar M, Martinez V (2015). Antibiotic-induced dysbiosis alters host-bacterial interactions and leads to colonic sensory and motor changes in mice. Gut Microbes.

[106] Obata T, Goto Y, Kunisawa J, Sato S, Sakamoto M, Setoyama H (2010). Indigenous opportunistic bacteria inhabit mammalian gut-associated lymphoid tissues and share a mucosal antibody-mediated symbiosis. Proc Natl Acad Sci U S A.

[107] Yang C, Mogno I, Contijoch EJ, Borgerding JN, Aggarwala V, Li Z (2020). Fecal IgA Levels Are Determined by Strain-Level Differences in Bacteroides ovatus and Are Modifiable by Gut Microbiota Manipulation. Cell Host Microbe.

[108] Lecuyer E, Rakotobe S, Lengline-Garnier H, Lebreton C, Picard M, Juste C (2014). Segmented filamentous bacterium uses secondary and tertiary lymphoid tissues to induce gut IgA and specific T helper 17 cell responses. Immunity.

[109] Qin W, Zhong X, Fan JM, Zhang YJ, Liu XR, Ma XY (2008). External suppression causes the low expression of the Cosmc gene in IgA nephropathy. Nephrol Dial Transplant.

[110] Uematsu S, Jang MH, Chevrier N, Guo Z, Kumagai Y, Yamamoto M (2006). Detection of pathogenic intestinal bacteria by Toll-like receptor 5 on intestinal CD11c+ lamina propria cells. Nat Immunol.

[111] Uematsu S, Akira S (2009). Immune responses of TLR5(+) lamina propria dendritic cells in enterobacterial infection. J Gastroenterol.

[112] Chen YY, Chen DQ, Chen L, Liu JR, Vaziri ND, Guo Y (2019). Microbiome-metabolome reveals the contribution of gut-kidney axis on kidney disease. J Transl Med.

[113] Huang Y, Zhou J, Wang S, Xiong J, Chen Y, Liu Y (2020). Indoxyl sulfate induces intestinal barrier injury through IRF1-DRP1 axis-mediated mitophagy impairment. Theranostics.

[114] Murphy EA, Velazquez KT, Herbert KM (2015). Influence of high-fat diet on gut microbiota: a driving force for chronic disease risk. Curr Opin Clin Nutr Metab Care.

[115] Luck H, Khan S, Kim JH, Copeland JK, Revelo XS, Tsai S (2019). Gut-associated IgA(+) immune cells regulate obesity-related insulin resistance. Nat Commun.

[116] Cheung CK, Barratt J (2015). Gluten and IgA nephropathy: you are what you eat?. Kidney Int.

[117] Papista C, Lechner S, Ben Mkaddem S, LeStang MB, Abbad L, Bex-Coudrat J (2015). Gluten exacerbates IgA nephropathy in humanized mice through gliadin-CD89 interaction. Kidney Int.

[118] Coppo R, Roccatello D, Amore A, Quattrocchio G, Molino A, Gianoglio B (1990). Effects of a gluten-free diet in primary IgA nephropathy. Clin Nephrol.

[119] Yap HK, Sakai RS, Woo KT, Lim CH, Jordan SC (1987). Detection of bovine serum albumin in the circulating IgA immune complexes of patients with IgA nephropathy. Clin Immunol Immunopathol.

[120] Sato M, Takayama K, Wakasa M, Koshikawa S (1987). Estimation of circulating immune complexes following oral challenge with cow's milk in patients with IgA nephropathy. Nephron.

[121] Kiryluk K, Li Y, Scolari F, Sanna-Cherchi S, Choi M, Verbitsky M (2014). Discovery of new risk loci for IgA nephropathy implicates genes involved in immunity against intestinal pathogens. Nat Genet.

[122] Lamas B, Richard ML, Leducq V, Pham HP, Michel ML, Da Costa G (2016). CARD9 impacts colitis by altering gut microbiota metabolism of tryptophan into aryl hydrocarbon receptor ligands. Nat Med.

[123] Sokol H, Conway KL, Zhang M, Choi M, Morin B, Cao Z (2013). Card9 mediates intestinal epithelial cell restitution, T-helper 17 responses, and control of bacterial infection in mice. Gastroenterology.

[124] Kunisawa J, Gohda M, Hashimoto E, Ishikawa I, Higuchi M, Suzuki Y (2013). Microbe-dependent CD11b+ IgA+ plasma cells mediate robust early-phase intestinal IgA responses in mice. Nat Commun.

[125] Fujikawa K, Miletic AV, Alt FW, Faccio R, Brown T, Hoog J (2003). Vav1/2/3-null mice define an essential role for Vav family proteins in lymphocyte development and activation but a differential requirement in MAPK signaling in T and B cells. J Exp Med.

[126] Vigorito E, Gambardella L, Colucci F, McAdam S, Turner M (2005). Vav proteins regulate peripheral B-cell survival. Blood.

[127] Ting SY, Martinez-Garcia E, Huang S, Bertolli SK, Kelly KA, Cutler KJ (2020). Targeted Depletion of Bacteria from Mixed Populations by Programmable Adhesion with Antagonistic Competitor Cells. Cell Host Microbe.

[128] Singh N, Gurav A, Sivaprakasam S, Brady E, Padia R, Shi H (2014). Activation of Gpr109a, receptor for niacin and the commensal metabolite butyrate, suppresses colonic inflammation and carcinogenesis. Immunity.

[129] Wahlstrom A, Sayin SI, Marschall HU, Backhed F (2016). Intestinal Crosstalk between Bile Acids and Microbiota and Its Impact on Host Metabolism. Cell Metab.

[130] Jacobson A, Lam L, Rajendram M, Tamburini F, Honeycutt J, Pham T (2018). A Gut Commensal-Produced Metabolite Mediates Colonization Resistance to Salmonella Infection. Cell Host Microbe.

[131] Huang J, Pearson JA, Peng J, Hu Y, Sha S, Xing Y (2020). Gut microbial metabolites alter IgA immunity in type 1 diabetes. JCI Insight.

[132] Song X, Sun X, Oh SF, Wu M, Zhang Y, Zheng W (2020). Microbial bile acid metabolites modulate gut RORgamma(+) regulatory T cell homeostasis. Nature.

[133] Frisbee AL, Petri WA Jr (2020). Considering the Immune System during Fecal Microbiota Transplantation for Clostridioides difficile Infection. Trends Mol Med.

[134] Paramsothy S, Nielsen S, Kamm MA, Deshpande NP, Faith JJ, Clemente JC (2019). Specific Bacteria and Metabolites Associated With Response to Fecal Microbiota Transplantation in Patients With Ulcerative Colitis. Gastroenterology.

[135] Sanders ME, Merenstein DJ, Reid G, Gibson GR, Rastall RA (2019). Probiotics and prebiotics in intestinal health and disease: from biology to the clinic. Nat Rev Gastroenterol Hepatol.

[136] Foligne B, Nutten S, Grangette C, Dennin V, Goudercourt D, Poiret S (2007). Correlation between *in vitro* and *in vivo* immunomodulatory properties of lactic acid bacteria. World J Gastroenterol.

[137] Flint HJ, Duncan SH, Scott KP, Louis P (2015). Links between diet, gut microbiota composition and gut metabolism. Proc Nutr Soc.

[138] Cybulsky AV, Walsh M, Knoll G, Hladunewich M, Bargman J, Reich H (2014). Canadian Society of Nephrology Commentary on the 2012 KDIGO clinical practice guideline for glomerulonephritis: management of glomerulonephritis in adults. Am J Kidney Dis.

[139] Schena FP, Manno C (2016). Intensive Supportive Care plus Immunosuppression in IgA Nephropathy. N Engl J Med.

[140] Lv J, Zhang H, Wong MG, Jardine MJ, Hladunewich M, Jha V (2017). Effect of Oral Methylprednisolone on Clinical Outcomes in Patients With IgA Nephropathy: The TESTING Randomized Clinical Trial. JAMA.

[141] Tang TT, Lv LL, Wang B, Cao JY, Feng Y, Li ZL (2019). Employing Macrophage-Derived Microvesicle for Kidney-Targeted Delivery of Dexamethasone: An Efficient Therapeutic Strategy against Renal Inflammation and Fibrosis. Theranostics.

[142] Yanagibashi T, Hosono A, Oyama A, Tsuda M, Suzuki A, Hachimura S (2013). IgA production in the large intestine is modulated by a different mechanism than in the small intestine: Bacteroides acidifaciens promotes IgA production in the large intestine by inducing germinal center formation and increasing the number of IgA+ B cells. Immunobiology.

[143] Edsbacker S, Andersson T (2004). Pharmacokinetics of budesonide (Entocort EC) capsules for Crohn's disease. Clin Pharmacokinet.

[144] Willis R, Seif AM, McGwin G Jr, Martinez-Martinez LA, Gonzalez EB, Dang N (2012). Effect of hydroxychloroquine treatment on pro-inflammatory cytokines and disease activity in SLE patients: data from LUMINA (LXXV), a multiethnic US cohort. Lupus.

[145] van den Borne BE, Dijkmans BA, de Rooij HH, le Cessie S, Verweij CL (1997). Chloroquine and hydroxychloroquine equally affect tumor necrosis factor-alpha, interleukin 6, and interferon-gamma production by peripheral blood mononuclear cells. J Rheumatol.

[146] Liu LJ, Yang YZ, Shi SF, Bao YF, Yang C, Zhu SN (2019). Effects of Hydroxychloroquine on Proteinuria in IgA Nephropathy: A Randomized Controlled Trial. Am J Kidney Dis.

[147] Bai L, Li J, Li H, Song J, Zhou Y, Lu R (2019). Renoprotective effects of artemisinin and hydroxychloroquine combination therapy on IgA nephropathy via suppressing NF-kappaB signaling and NLRP3 inflammasome activation by exosomes in rats. Biochem Pharmacol.

[148] Merrill JT, Wallace DJ, Wax S, Kao A, Fraser PA, Chang P (2018). Efficacy and Safety of Atacicept in Patients With Systemic Lupus Erythematosus: Results of a Twenty-Four-Week, Multicenter, Randomized, Double-Blind, Placebo-Controlled, Parallel-Arm, Phase IIb Study. Arthritis Rheumatol.

[149] Kim YG, Alvarez M, Suzuki H, Hirose S, Izui S, Tomino Y (2015). Pathogenic Role of a Proliferation-Inducing Ligand (APRIL) in Murine IgA Nephropathy. PLoS One.

[150] Myette JR, Kano T, Suzuki H, Sloan SE, Szretter KJ, Ramakrishnan B (2019). A Proliferation Inducing Ligand (APRIL) targeted antibody is a safe and effective treatment of murine IgA nephropathy. Kidney Int.

[151] Edwards JC, Szczepanski L, Szechinski J, Filipowicz-Sosnowska A, Emery P, Close DR (2004). Efficacy of B-cell-targeted therapy with rituximab in patients with rheumatoid arthritis. N Engl J Med.

[152] Fervenza FC, Abraham RS, Erickson SB, Irazabal MV, Eirin A, Specks U (2010). Rituximab therapy in idiopathic membranous nephropathy: a 2-year study. Clin J Am Soc Nephrol.

[153] Lafayette RA, Canetta PA, Rovin BH, Appel GB, Novak J, Nath KA (2017). A Randomized, Controlled Trial of Rituximab in IgA Nephropathy with Proteinuria and Renal Dysfunction. J Am Soc Nephrol.

[154] Hartono C, Chung M, Perlman AS, Chevalier JM, Serur D, Seshan SV (2018). Bortezomib for Reduction of Proteinuria in IgA Nephropathy. Kidney Int Rep.

[155] Zhong Z, Li H, Zhong H, Zhou T (2019). Clinical efficacy and safety of rituximab in lupus nephritis. Drug Des Devel Ther.

[156] Bologna L, Gotti E, Manganini M, Rambaldi A, Intermesoli T, Introna M (2011). Mechanism of action of type II, glycoengineered, anti-CD20 monoclonal antibody GA101 in B-chronic lymphocytic leukemia whole blood assays in comparison with rituximab and alemtuzumab. J Immunol.

[157] Du FH, Mills EA, Mao-Draayer Y (2017). Next-generation anti-CD20 monoclonal antibodies in autoimmune disease treatment. Auto Immun Highlights.

[158] Miyazaki T, Hirokami Y, Matsuhashi N, Takatsuka H, Naito M (1999). Increased susceptibility of thymocytes to apoptosis in mice lacking AIM, a novel murine macrophage-derived soluble factor belonging to the scavenger receptor cysteine-rich domain superfamily. J Exp Med.

[159] Haruta I, Kato Y, Hashimoto E, Minjares C, Kennedy S, Uto H (2001). Association of AIM, a novel apoptosis inhibitory factor, with hepatitis via supporting macrophage survival and enhancing phagocytotic function of macrophages. J Biol Chem.

[160] Martinez VG, Escoda-Ferran C, Tadeu Simoes I, Arai S, Orta Mascaro M, Carreras E (2014). The macrophage soluble receptor AIM/Api6/CD5L displays a broad pathogen recognition spectrum and is involved in early response to microbial aggression. Cell Mol Immunol.

[161] Irabu H, Shimizu M, Kaneko S, Inoue N, Mizuta M, Tasaki Y (2020). Apoptosis inhibitor of macrophage as a biomarker for disease activity in Japanese children with IgA nephropathy and Henoch-Schonlein purpura nephritis. Pediatr Res.

[162] Takahata A, Arai S, Hiramoto E, Kitada K, Kato R, Makita Y (2020). Crucial Role of AIM/CD5L in the Development of Glomerular Inflammation in IgA Nephropathy. J Am Soc Nephrol.

[163] Vidarsson G, Overbeeke N, Stemerding AM, van den Dobbelsteen G, van Ulsen P, van der Ley P (2005). Working mechanism of immunoglobulin A1 (IgA1) protease: cleavage of IgA1 antibody to Neisseria meningitidis PorA requires de novo synthesis of IgA1 Protease. Infect Immun.

[164] Senior BW, Woof JM (2005). The influences of hinge length and composition on the susceptibility of human IgA to cleavage by diverse bacterial IgA1 proteases. J Immunol.

[165] Long S, Phan E, Vellard MC (2010). The expression of soluble and active recombinant Haemophilus influenzae IgA1 protease in E. coli. J Biomed Biotechnol.

[166] Lechner SM, Abbad L, Boedec E, Papista C, Le Stang MB, Moal C (2016). IgA1 Protease Treatment Reverses Mesangial Deposits and Hematuria in a Model of IgA Nephropathy. J Am Soc Nephrol.

[167] Chen A, Yang SS, Lin TJ, Ka SM (2018). IgA nephropathy: clearance kinetics of IgA-containing immune complexes. Semin Immunopathol.

[168] van Zandbergen G, van Kooten C, Mohamad NK, Reterink TJ, de Fijter JW, van de Winkel JG (1998). Reduced binding of immunoglobulin A (IgA) from patients with primary IgA nephropathy to the myeloid IgA Fc-receptor, CD89. Nephrol Dial Transplant.

[169] van Egmond M, van Garderen E, van Spriel AB, Damen CA, van Amersfoort ES, van Zandbergen G (2000). FcalphaRI-positive liver Kupffer cells: reappraisal of the function of immunoglobulin A in immunity. Nat Med.

[170] Gormly AA, Smith PS, Seymour AE, Clarkson AR, Woodroffe AJ (1981). IgA glomerular deposits in experimental cirrhosis. Am J Pathol.

[171] Melvin T, Burke B, Michael AF, Kim Y (1983). Experimental IgA nephropathy in bile duct ligated rats. Clin Immunol Immunopathol.

[172] Rosenblad T, Rebetz J, Johansson M, Bekassy Z, Sartz L, Karpman D (2014). Eculizumab treatment for rescue of renal function in IgA nephropathy. Pediatr Nephrol.

[173] Floege J, Feehally J (2013). Treatment of IgA nephropathy and Henoch-Schonlein nephritis. Nat Rev Nephrol.

[174] Kamiyama T, Moriyama T, Kumon S, Karasawa K, Nitta K (2019). The beneficial effects of renin-angiotensin system inhibitors (RASI) on IgA nephropathy with tubulointerstitial lesions categorized by Oxford classification. Clin Exp Nephrol.

[175] Tang SC, Lin M, Tam S, Au WS, Ma MK, Yap DY (2012). Aliskiren combined with losartan in immunoglobulin A nephropathy: an open-labeled pilot study. Nephrol Dial Transplant.

[176] McAdoo S, Tam FWK (2018). Role of the Spleen Tyrosine Kinase Pathway in Driving Inflammation in IgA Nephropathy. Semin Nephrol.

[177] McAdoo SP, Reynolds J, Bhangal G, Smith J, McDaid JP, Tanna A (2014). Spleen tyrosine kinase inhibition attenuates autoantibody production and reverses experimental autoimmune GN. J Am Soc Nephrol.

[178] Pamuk ON, Can G, Ayvaz S, Karaca T, Pamuk GE, Demirtas S (2015). Spleen tyrosine kinase (Syk) inhibitor fostamatinib limits tissue damage and fibrosis in a bleomycin-induced scleroderma mouse model. Clin Exp Rheumatol.

[179] Nagai K, Miyoshi M, Kake T, Fukushima N, Matsuura M, Shibata E (2013). Dual involvement of growth arrest-specific gene 6 in the early phase of human IgA nephropathy. PLoS One.

